# Transcriptomic Analysis of Salivary Glands of *Ornithodoros brasiliensis* Aragão, 1923, the Agent of a Neotropical Tick-Toxicosis Syndrome in Humans

**DOI:** 10.3389/fphys.2021.725635

**Published:** 2021-08-06

**Authors:** Jose Reck, Anelise Webster, Bruno Dall’Agnol, Ronel Pienaar, Minique H. de Castro, Jonathan Featherston, Ben J. Mans

**Affiliations:** ^1^Instituto de Pesquisas Veterinárias Desidério Finamor (IPVDF), Eldorado do Sul, Brazil; ^2^Epidemiology, Parasites and Vectors, Agricultural Research Council, Onderstepoort Veterinary Research, Pretoria, South Africa; ^3^Agricultural Research Council, Biotechnology Platform, Pretoria, South Africa; ^4^Department of Veterinary Tropical Diseases, Vector and Vector-borne Disease Research Programme, University of Pretoria, Pretoria, South Africa; ^5^Department of Life and Consumer Sciences, University of South Africa, Pretoria, South Africa

**Keywords:** RNA, next-generation sequencing, argasid, nymph, secretory proteins

## Abstract

Tick salivary glands produce and secrete a variety of compounds that modulate host responses and ensure a successful blood meal. Despite great progress made in the identification of ticks salivary compounds in recent years, there is still a paucity of information concerning salivary molecules of Neotropical argasid ticks. Among this group of ticks, considering the number of human cases of parasitism, including severe syndromes and hospitalization, *Ornithodoros brasiliensis* can be considered one of the major Neotropical argasid species with impact in public health. Here, we describe the transcriptome analysis of *O. brasiliensis* salivary glands (ObSG). The transcriptome yielded ~14,957 putative contigs. A total of 368 contigs were attributed to secreted proteins (SP), which represent approximately 2.5% of transcripts but ~53% expression coverage transcripts per million. Lipocalins are the major protein family among the most expressed SP, accounting for ~16% of the secretory transcripts and 51% of secretory protein abundance. The most expressed transcript is an ortholog of TSGP4 (tick salivary gland protein 4), a lipocalin first identified in *Ornithodoros kalahariensis* that functions as a leukotriene C_4_ scavenger. A total of 55 lipocalin transcripts were identified in ObSG. Other transcripts potentially involved in tick-host interaction included as: basic/acid tail secretory proteins (second most abundant expressed group), serine protease inhibitors (including Kunitz inhibitors), 5' nucleotidases (tick apyrases), phospholipase A_2_, 7 disulfide bond domain, cystatins, and tick antimicrobial peptides. Another abundant group of proteins in ObSG is metalloproteases. Analysis of these major protein groups suggests that several duplication events after speciation were responsible for the abundance of redundant compounds in tick salivary glands. A full mitochondrial genome could be assembled from the transcriptome data and confirmed the close genetic identity of the tick strain sampled in the current study, to a tick strain previously implicated in tick toxicoses. This study provides novel information on the molecular composition of ObSG, a Brazilian endemic tick associated with several human cases of parasitism. These results could be helpful in the understanding of clinical findings observed in bitten patients, and also, could provide more information on the evolution of Neotropical argasids.

## Introduction

Ticks (Ixodida) are composed of three families, the hard (Ixodidae), soft (Argasidae), and monotypic Nuttalliellidae (Guglielmone et al., 2010). The Ixodida is obligate blood-feeding ecto-parasites that secrete a cocktail of bioactive salivary gland-derived components during feeding, to counteract the vertebrate host’s defense mechanisms, such as blood-clotting, platelet aggregation, and inflammation (Mans, 2019). Ixodids in all life stages feed for prolonged periods that may last for days to weeks during which hundreds to thousands of salivary gland proteins are differentially secreted, presumably to evade the host’s changing immune responses, but also to adapt to a changing feeding environment due to the host’s healing responses (Francischetti et al., 2009). Argasids show much more diverse feeding behavior in different life stages. In the larval stages, some species molt to nymphs without feeding, other species feed rapidly within minutes, while some species feed for prolonged periods of days-weeks resembling ixodids. In the nymphal stages, instars from some species do not feed, but molt to the next developmental stage, while females from some species also do not feed, although the majority of species requires a blood meal for successful oviposition (Hoogstraal, 1985).

In the case of *Ornithodoros brasiliensis* Aragão, 1923, the larvae molt to nymphs without feeding, while nymphal stages fed within 25–35 min similar to adults (Ramirez et al., 2016). Bites by this tick species result in toxicoses most probably due to injection of salivary gland components into the feeding site (Reck et al., 2011, 2013a, 2014; Dall’Agnol et al., 2019). Toxicoses by the “mouro tick” exhibit as erythemic and swollen lesions, hyperemia of oral/ocular mucosa pruritus, and tachypnea and slow wound-healing processes, and in recent years, cases of human hospitalization after tick bite have been reported (Reck et al., 2011, 2013a,b, 2014; Dall’Agnol et al., 2019). It has been shown that salivary gland homogenates can inhibit wound healing and endothelial cell proliferation *in vitro* (Reck et al., 2013b). To date, little is known about the salivary gland protein composition of this tick species that would help to explain these effects or symptoms.

Salivary gland transcriptome sequencing is a useful tool to generate catalogs of salivary gland-derived transcripts and has been important in the description of salivary gland protein sequence diversity (Mans, 2020). As such, characterization of the salivary gland transcriptome of *O. brasiliensis* would create an important resource to elucidate the molecular mechanisms behind mouro tick toxicoses. Argasid salivary gland transcriptomes have been characterized in detail using conventional cDNA library Sanger sequencing for *Antricola delacruzi* Estrada-Peña, Barros-Battesti, and Venzal, 2004 (Ribeiro et al., 2012), *Argas monolakensis* Schwan, Corwin, and Brown, 1992 (Mans et al., 2008a), *Ornithodoros coriaceus* Koch, 1844 (Francischetti et al., 2008a), and *Ornithodoros parkeri* Cooley, 1936 (Francischetti et al., 2008b). Argasid salivary gland transcriptomes have also been characterized using next-generation sequencing and assembly strategies for *Ornithodoros moubata* (Murray, 1877) (Pérez-Sánchez et al., 2021), *Ornithodoros erraticus* (Lucas, 1849) (Oleaga et al., 2021), and *Ornithodoros rostratus* Aragão, 1911 (Araujo et al., 2019). The salivary gland transcriptome of *O. brasiliensis* has not been described yet. The aim of the current study was to sequence the nymphal salivary gland transcriptome of *O. brasiliensis* ticks using next-generation sequencing.

## Materials and Methods

### Tick Collection and RNA Extraction

Nymphs of *O. brasiliensis* were collected in the field from a site previously implicated in tick parasitism of travelers in Brazil (Dall’Agnol et al., 2019) and maintained unfed in the laboratory for ~2 months before dissection. Salivary glands were dissected from 10 nymphs and placed in RNA later before storing at −70°C. Total RNA was extracted using the RNeasy Protect Mini Kit (QIAGEN Group). Briefly, glands were suspended in 500 μl RLT buffer and disrupted by 10X passage through an 18G needle followed by 10X passage using a 24G needle. Residual genomic DNA was removed with DNase I digestion. Total RNA quantification was performed using the Qubit fluorimeter 2.0 (Life Technologies, Carlsbad, CA).

### Library Construction and Next-Generation Sequencing

For library preparation, 1.0 μg purified total RNA was used with the TruSeq stranded mRNA sample preparation kit (Illumina, San Diego, CA). Poly-A mRNA was isolated, fragmented (for 3 min), and converted to double-stranded cDNA, adapters ligated, and PCR amplified for 12 cycles. Amplified bands were size selected from 450 to 1,200 bp. Bands were excised, purified, and sequenced using the Illumina MiSeq system (300 bp×300 bp). Raw sequence reads were submitted to GenBank under BioProject PRJNA719007 with small read archive accession number: SRR14139641.

### Transcriptome Assembly

Raw Illumina reads were quality trimmed (0.001 quality limit) and TruSeq adapters removed using CLC Genomics Workbench. Reads were imported as single or paired end reads. The paired end reads were merged to produce a merged dataset (Merged), while the single reads were used as unpaired (Single) and to produce a single-merged dataset (SM). Duplicates were also removed from these datasets to produce three duplicate removed datasets (Mddup, Sddup, and SMddup; Pienaar et al., 2021). These datasets were used to assemble the transcriptome using Trinity v2.4.0 and CLC Genomics Workbench v 20.0. Trinity was used with default parameter settings of kmer size 25. For CLC Genomics Workbench, kmer sizes were used in step sizes of 5 starting at 15 up to 60 and an additional assembly using kmer 64 (11 assemblies) with assembly parameters: mismatch cost-2, insertion cost-3, deletion cost-3, length fraction-0.9, similarity-0.9, minimum contig length-240, kmer size-variable, and bubble size-automatic. Given the different dataset structures used, a total of 72 assemblies were produced.

### Extraction of the Mitochondrial Genome and Phylogenetic Analysis

The mitochondrial genome was identified in the assemblies by BLASTN analysis (Altschul et al., 1993), using the previously published mitochondrial genome for *O. brasiliensis* (Burger et al., 2014). The mitochondrial genome was annotated using the MITOS server to identify tRNA genes (Bernt et al., 2013). Protein coding and rRNA genes were identified using BLAST analysis (Altschul et al., 1993). The translated COI, CYTB, ND1, ND2, and ND4 proteins were used for phylogenetic analysis as previously described (Mans et al., 2015, 2019, 2021).

### Extraction of CDS and Quality Assessment

Open reading frames (ORFs) were extracted using a Perl-script and chimeric and duplicate sequences removed by clustering at 90% identity using CD-HIT (Li and Godzik, 2006). The single dataset was mapped against the clustered ORFs using CLC Genomics Workbench and ORFs with TPM>1 (transcripts per million) were selected. BLASTX analysis against the ACARI database reduced the dataset further by selecting ORFs with E-values below 0.004 for further analysis. The transcriptome quality was measured for accuracy, completeness, contiguity, and chimerism using the Benchmarking Universal Single-Copy Orthologs (BUSCO; Simao et al., 2015). The final set of ORFs was submitted to GenBank under BioProject PRJNA719007.

### Bioinformatic Analysis of the Transcriptome

To identify potential secretory peptides translated ORFs were submitted to SignalP (Petersen et al., 2011), while TMHMM and Phobius (Krogh et al., 2001; Kall et al., 2004) were used to identify membrane proteins. Potential housekeeping and secretory proteins were identified by BLASTP analysis against an ACARI database annotated using the KEGG database and GhostKOALA (Kanehisa et al., 2016a,b), the TSFAM database (Ribeiro and Mans, 2020), and an in-house annotation of secretory protein families (de Castro et al., 2016). To identify functional orthologs of proteins with experimentally verified functions, BLASTP analysis of secretory proteins was performed against the NCBI non-redundant database before phylogenetic analysis was performed to confirm clustering in functional clades with high bootstrap support. Protein families were aligned using MAFFT (Katoh and Standley, 2013), and maximum-likelihood analysis performed using IQ-Tree2 v 1.6.12 (Minh et al., 2020) with a standard 1,000,000 bootstraps.

## Results and Discussion

### Transcriptome Assembly

**Figure 1:**
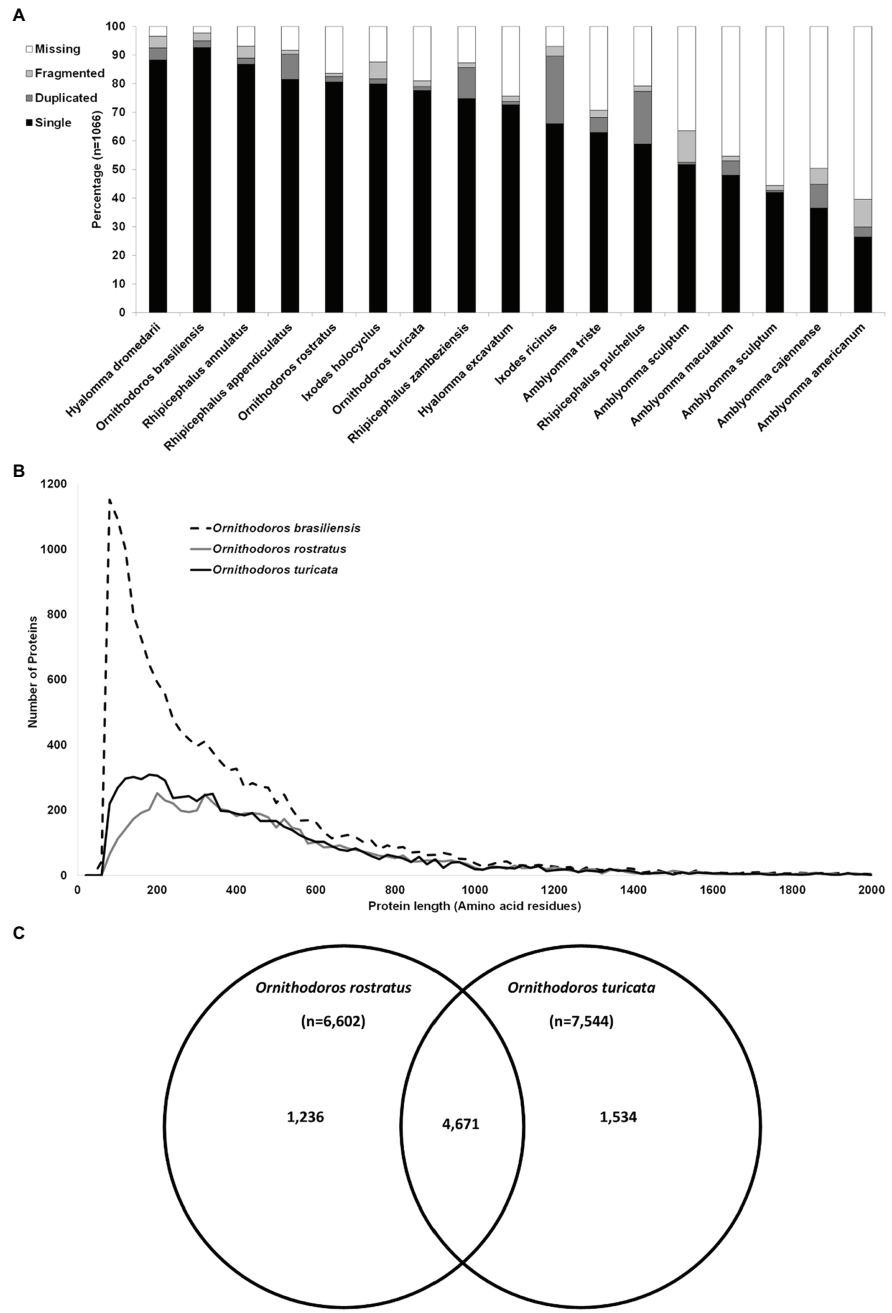
Quality assessment of the transcriptome. **(A)** Comparison of the BUSCO analysis of all published salivary gland transcriptomes and *Ornithodoros brasiliensis*. The species are indicated on the horizontal axis, while the percentage of single complete, duplicated complete, missing, and fragmented is indicated on the vertical axis. Transcriptomes used in the comparison are as: *H. dromedarii* (Bensaoud et al., 2018), *R. annulatus* (Antunes et al., 2019), *R. appendiculatus* (de Castro et al., 2016), *O. rostratus* (Araujo et al., 2019), *I. holocyclus* (Rodriguez-Valle et al., 2018), *O. turicata* (Bourret et al., 2019), *R. zambeziensis* (de Castro et al., 2017), *H. excavatum* (Ribeiro et al., 2017), *I. ricinus* (Schwarz et al., 2013, 2014), *A. triste* (Garcia et al., 2014), *R. pulchellus* (Tan et al., 2015), *A. sculptum* (Moreira et al., 2017), *A. maculatum* (Karim et al., 2011), *A. sculptum* (Esteves et al., 2017), *A. cajennense* (Garcia et al., 2014), and *A. americanum* (Karim and Ribeiro, 2015). **(B)** Comparison of protein sequence length in amino acid residues presented up to 2,000 residues against the number of proteins. Proteins were binned in windows of 20 based on protein length. **(C)** Reciprocal best hit analysis of the transcriptome of *O. brasiliensis* (*n* = 14,957) against two closely related Neotropic and Nearctic tick species. Indicated are orthologs shared uniquely between species pairs or shared between all three species.

**Figure 2:**
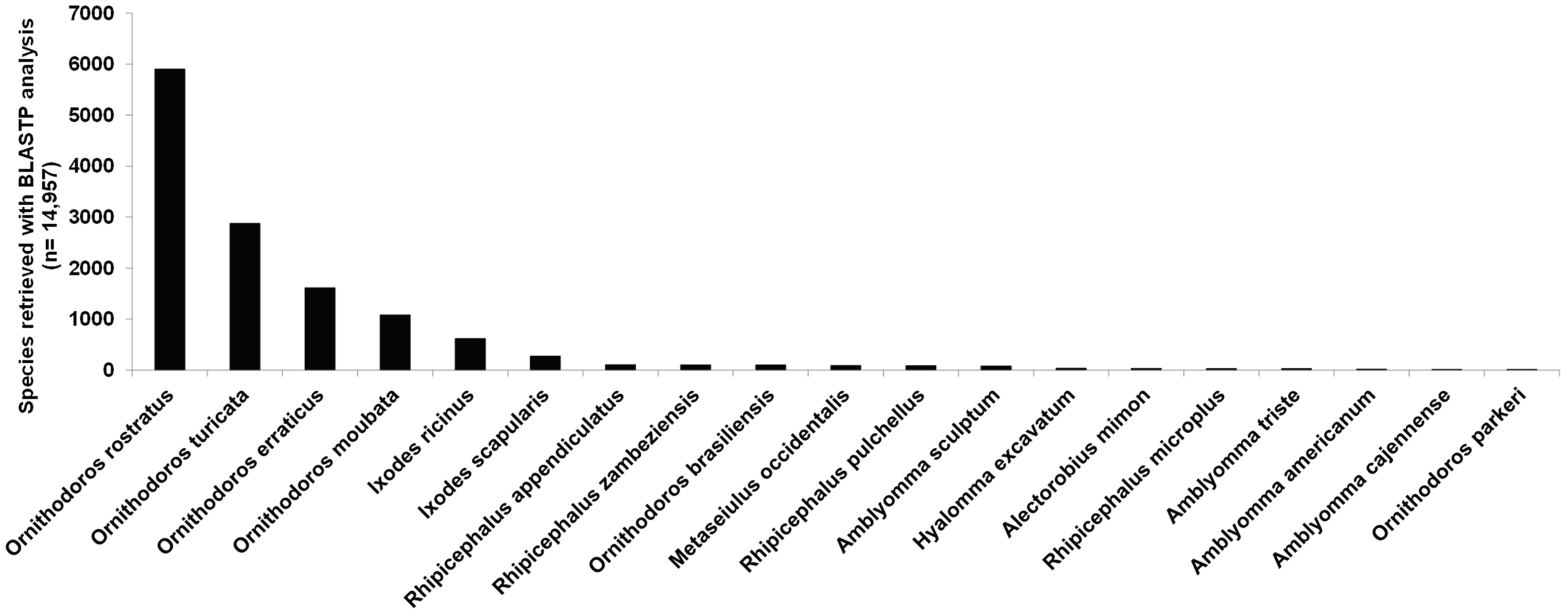
BLASTP analysis of 14,957 contigs against the ACARI database. Species with more than 10 hits are indicated.

A total of 58,490,632 paired reads were generated that yielded 2,285,451 merged reads (Merged) after quality trimming and merging and 10,505,566 single reads (Single) after quality trimming, resulting in a combined 12,791,017 reads (SM). Removal of duplicate reads resulted in 1,301,316 merged reads (Mddup), 6,841,083 single reads (Sddup), and combined 8,142,399 reads (SMddup). Assembly resulted in 100,397 contigs after clustering with CDHIT that was further reduced to 44,052 contigs with TPM>1. This resulted in 16,908 contigs with E-values >0.004 and 14,957 contigs after manual curation. BUSCO analysis indicated 95.0% completeness with 92.6% as single genes, 2.4% as duplicated, 2.7% fragmented, and 2.3% missing from a set of 1,066 conserved genes. This compares well with other tick transcriptomes sequenced thus far (Figure 1A). Comparison to two other soft tick salivary gland transcriptomes (*O. rostratus* and *Ornithodoros turicata* which belong to the Neotropic and Nearctic *Pavlovskyella*), for which protein sequence data are available in the public repositories, indicates that *O. brasiliensis* generally has longer contigs although it also has higher numbers of short contigs, which may indicate that some contigs may be truncated (Figure 1B). Reciprocal best hit analysis indicated that *O. brasiliensis* shares 7,441 orthologs in total with these transcriptomes (Figure 1C). This can be considered a minimum, since this is limited by the numbers of contigs submitted for the other transcriptomes, i.e., *O. rostratus* (*n* = 6,602) and *O. turicata* (*n* = 7,544). As such, the percentage of orthologs shared for each transcriptome is 89% of *O. rostratus* and 82% of *O. turicata*. These measures were taken to indicate a well-represented high quality transcriptome. BLASTP analysis of ACARI database using the transcriptome retrieved as highest number of hits, proteins from related soft ticks, such as *O. rostratus*, *O. turicata*, *O. erraticus*, and *O. moubata* (Figure 2). This may be expected but is also a good measure of transcriptome quality.

### Mitochondrial Genome Analysis

**Figure 3:**
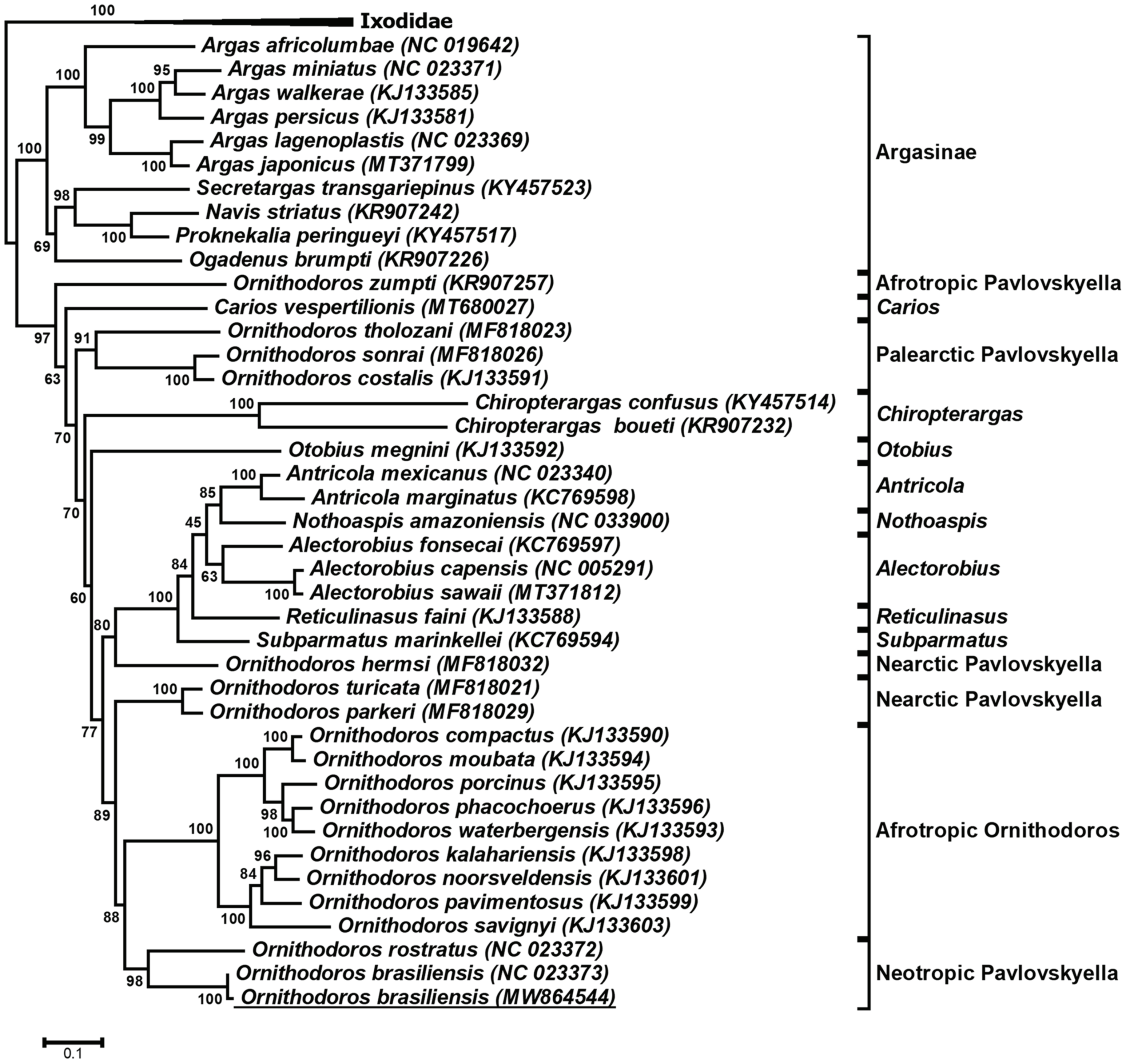
Phylogenetic analysis of argasid mitochondrial genes. Indicated is a maximum-likelihood tree of the protein coding genes COI, CYTB, ND1, ND2, and ND4 of the mitochondrial genome. Bootstrap values for 1,000,000 bootstraps are indicated. The tree was rooted with the Ixodidae. The mitochondrial genome obtained in the current study is underlined.

Previously, *O. brasiliensis* ticks shown to cause toxicoses and for which a mitochondrial genome was sequenced were collected at a site distant from the current study (Reck et al., 2013a; Burger et al., 2014). Since the collection sites were approximately 60 km apart, the question was raised regarding the genetic relationship of the ticks collected in this study and that of the mitochondrial genome previously sequenced for this species. The full-length mitochondrial genome was assembled in the transcriptome and was 99% identical to the previously sequenced genome and was deposited under accession number MW864544. Phylogenetic analysis indicated that both genomes cluster together with high support in a clade shared with *O. rostratus* in what has been described as the Neotropic *Pavlovskyella* clade (Figure 3). The sister-clade with high bootstrap support is that of the Afrotropic *Ornithodoros* sensu stricto group suggesting as previously indicated that these clades probably diverged during continental breakup of Gondwanaland ~127 MYA (Mans et al., 2019). This places the transcriptome within a phylogenetic context for comparative analysis and suggests that the transcriptomes of Afrotropic *Ornithodoros* and Neotropic *Pavlovskyella* should share extensive similarities with regard to orthologs and function. In addition, full-length 18S and 28S rRNA sequences were retrieved from the study and were deposited under GenBank accession numbers MW857182 and MW877711, respectively. These commonly used phylogenetic markers can therefore also be retrieved from transcriptome sequencing projects.

### Composition of the Transcriptome

**Figure 4:**
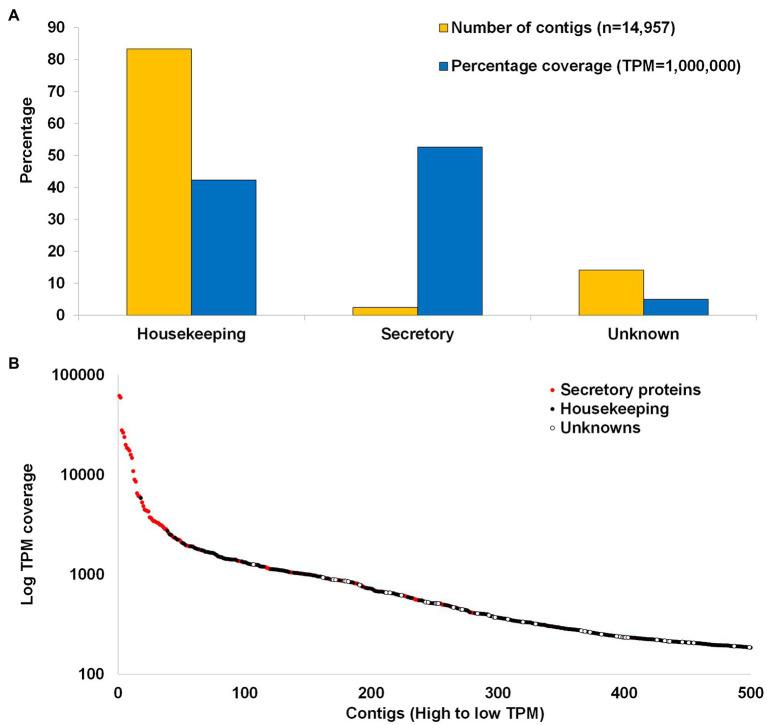
Summary of transcriptome. **(A)** Comparison of the housekeeping, secretory, and unknown classes of contigs with regard to number of contigs contained in each class and the cumulative coverage of each class. **(B)** The first 500 contigs with the highest TPM coverage. Red dots indicate secretory proteins and black dots indicate housekeeping while white dots indicate unknown proteins.

The transcriptome can be divided into housekeeping (*n* = 12,471), secretory (*n* = 368), and unknown categories (*n* = 2,118; Figure 4A). Housekeeping proteins are defined as all proteins not part of the secretory class that is involved in general cellular or organismal housekeeping functions, while secretory proteins are defined as those with secretory peptides, considered to be secreted into the feeding site during feeding, while unknown proteins refer to proteins with significant BLASTP hits in the database, but which has no annotation. These classifications have been used in all tick transcriptome studies dating back to Ribeiro et al. (2006). Housekeeping proteins account for ~83.3% of the transcriptome, secretory proteins for ~2.5%, and unknown proteins for ~14.1%. Reads mapped back to the transcriptome indicate that housekeeping proteins account for ~42% of the coverage, secretory proteins for ~53%, and unknowns for ~5%. This would suggest that secretory proteins are present at higher concentrations in the salivary glands relative to the housekeeping proteins. To corroborate this, the first 39 proteins with highest coverage are secretory proteins and comprise 43% of the total TPM coverage (80% of the secretory protein contribution; Figure 4B). The high abundance of secretory proteins was previously observed in soft tick salivary glands and may be explained by the fact that soft ticks synthesize secretory proteins and store them in large secretory granules that occupy most of the space in the salivary gland cells until secretion (Mans and Neitz, 2004; Mans et al., 2004). Abundance of secretory transcripts was also observed in conventional Sanger sequenced argasid salivary gland transcriptomes, as well as in those sequenced with next-generation sequencing technologies (Francischetti et al., 2008a,b; Mans et al., 2008a; Araujo et al., 2019).

### Housekeeping Proteins

**Figure 5:**
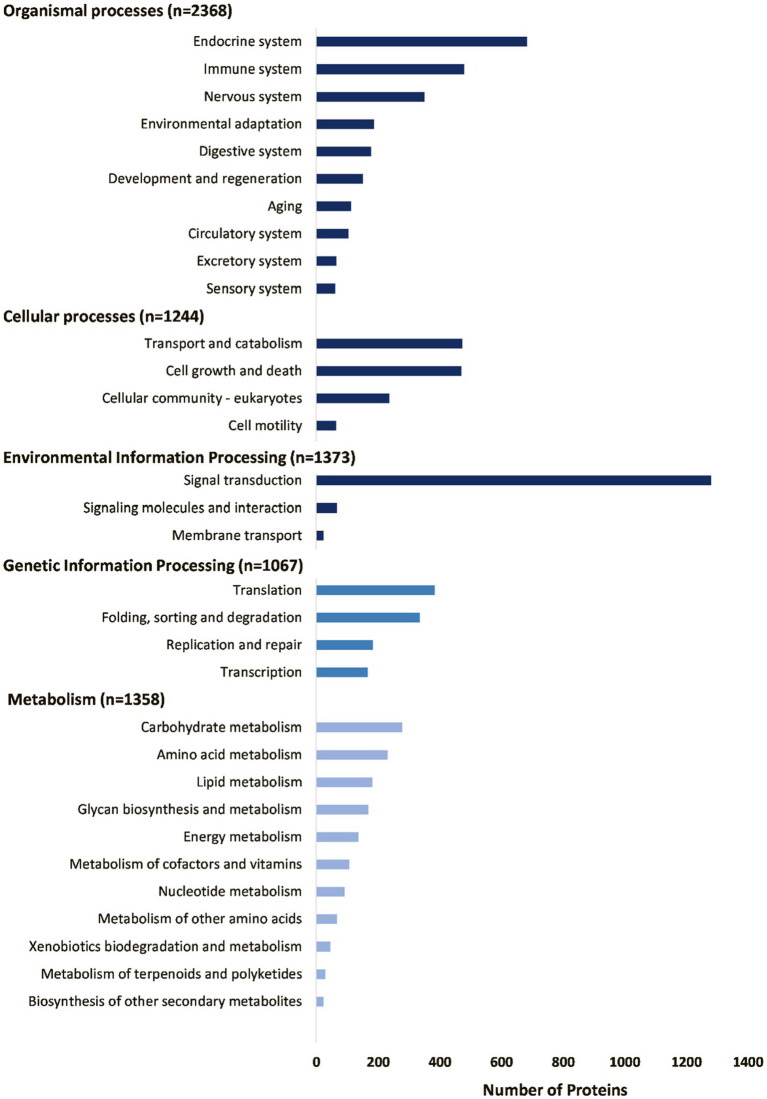
Summary of housekeeping proteins as annotated by the GhostKOALA search of the KEGG database.

**Figure 6:**
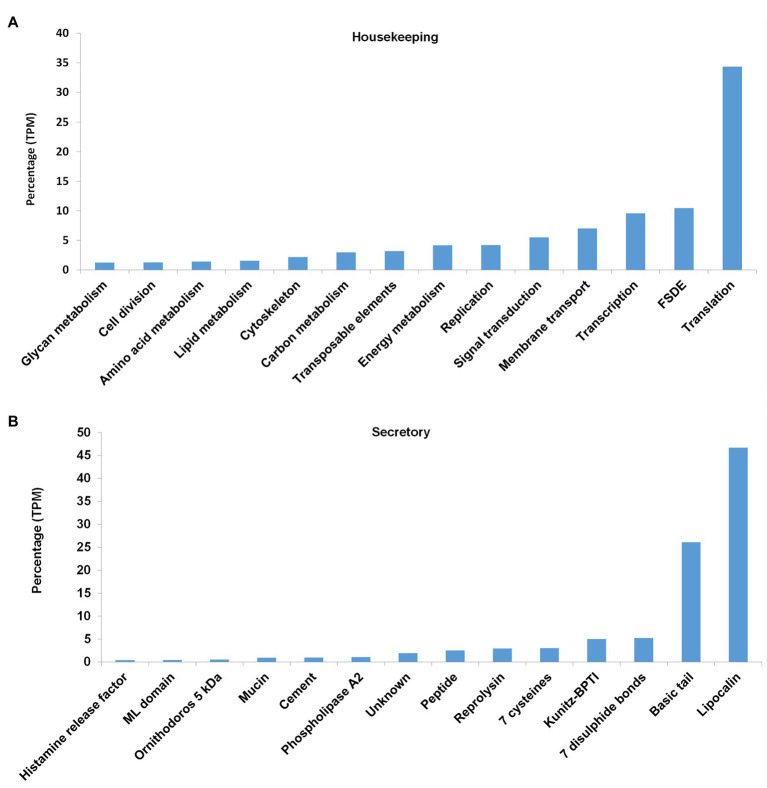
Abundance of housekeeping and secretory proteins expressed as percentage of total coverage (TPM). **(A)** For the housekeeping proteins, the classes with >1% total coverage (~90% of total coverage) are indicated. FSDE: folding, sorting, degradation, and excretion. **(B)** For the secretory proteins, the classes with >0.2% total coverage (~98% of total coverage) are indicated.

The housekeeping proteins can be divided into various functional classes as classified by the KEGG database (Figure 5). These include proteins involved in metabolism, protein synthesis (transcription and translation), protein folding, sorting, degradation and excretion (FSDE), environmental sensing processes, such as signal transduction systems, cellular processes, such as cell growth, death, and motility, and organismal processes, such as the circulatory, developmental, digestive, endocrine, excretory, immune, nervous, and sensory systems. Signal transduction processes are notably enriched with regard to number of proteins. The most abundant housekeeping classes include those involved in protein synthesis (transcription and translation), FSDE (Figure 6A), that cumulatively account for 54% of TPM coverage for housekeeping proteins. This may be expected for an organ, such as salivary glands whose primary function is the synthesis, folding, and sorting of secretory proteins.

Some housekeeping functions with specific reference to tick biology may be highlighted. Of ~270 proteins identified in vertebrates to function as part of the protein secretory pathway (Gutierrez et al., 2020), 216 orthologs could be identified with confidence (Supplementary Table 1). The reason for many of those not identified may be due to multiple isoforms in the vertebrate secretory system which may be only represented by single proteins in ticks. This suggests that a large portion of the secretory system is present in the current transcriptome and is also conserved in ticks. This may be expected since the secretory pathway may be considered one of the Lineages of Life processes conserved in all Metazoa (Mans et al., 2016). Similarly, several orthologs of proteins previously implicated in neuronal control of salivary gland secretion in ticks (Šimo et al., 2014) were identified (Supplementary Table 2). This included hormones, such as bursicon, crustacean hyperglycemic hormone, eclosion hormone, elevenin, insulin, and orcokinin, as well as hormone receptors, such as allatostatin receptor, calcitonin gene-related peptide type 1 receptor, corazonin receptor 1, dopamine receptor 1, dopamine receptor 2-like, dopamine D2-like receptor, elevenin receptor 2, insulin-like growth factor 1 receptor, tachykinin-like peptides receptor, and periviscerokinin/Cap2b receptor.

It was previously indicated that the heme biosynthesis pathway is incomplete in ticks with ixodids lacking hemA-hemE, but possess hemF-hemH (Braz et al., 1999; Mans et al., 2016; Perner et al., 2016). Argasids possessed hemB and hemF-hemH (Mans et al., 2016). BLASTP analysis using hemA-hemH for *Metaseiulus occidentalis* indicated orthologs for hemB, hemF, and hemG, consistent with previous observations for argasids.

### Secretory Proteins

**Figure 7:**
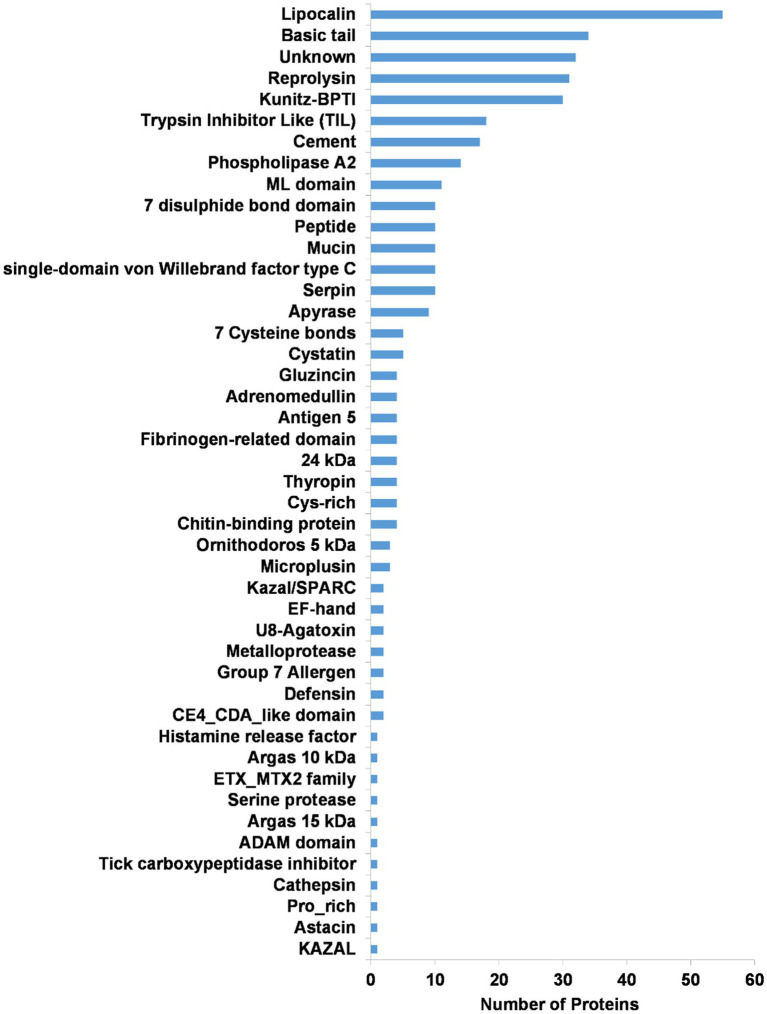
Summary of the secretory protein families found in the salivary gland transcriptome of *O. brasiliensis*. Indicated are numbers of proteins for each class.

Secretory protein families identified in the transcriptome included the majority of protein families generally found in tick salivary glands (Figures 6B, 7). The most abundant protein families in terms of sequence coverage (TPM) were the lipocalin, basic tail secretory family (BTSP), 7 disulfide bond domain (7DB), Kunitz-BPTI domains, 7 cysteine domain, and reprolysin, comprising ~98% of the total (Figure 6B). The lipocalin, basic tail, reprolysin, and Kunitz-BPTI families were also the most abundant in terms of the number of family members (Figure 7). This has previously been observed in soft tick salivary gland transcriptomes (Francischetti et al., 2008a,b; Mans et al., 2008a; Araujo et al., 2019). In addition, a number of peptides with secretory signals but no BLASTP hits were also identified as unknown proteins.

### Potential Proteins Functional at the Tick-Host Interface

Approximately 120 protein functions involved at the tick-host interface has been experimentally validated (Mans et al., 2019). BLASTP analysis using these proteins found 73 potential orthologs for 13 functions (Table 1). This includes the 5' nucleotidase family member apyrase that inhibits platelet aggregation by hydrolyzing ADP (Ribeiro et al., 1991; Mans et al., 1998; Stutzer et al., 2009).

**Table 1:** Orthologs for proteins with experimentally confirmed functions.

Protein	Function/target	Protein family	Orthologs in transcriptome
Apyrase	ATP/ADP hydrolysis	5' Nucleotidase	Obras3069, Obras3096, Obras3118
Moubatin	Leukotriene B_4_ binding	Lipocalin	Obras9831, Obras10708, Obras11692, Obras11697
TSGP1	Biogenic amine binding	Lipocalin	Obras8892, Obras9626, Obras11694, Obras11530, Obras11349, Obras10580, Obras10207
TSGP4	Leukotriene C_4_ binding	Lipocalin	Obras8357, Obras9228, Obras9233, Obras9535, Obras9761, Obras10025, Obras10029, Obras10067, Obras10069, Obras10481, Obras11033, Obras12035
Savicalin	Antimicrobial	Lipocalin	Obras10004
Defensin	Antimicrobial	Defensin	Obras14234, Obras33514
Microplusin	Antimicrobial	Microplusin	Obras16239, Obras16257, Obras20108
TIL domain	Antimicrobial	TIL domain	Obras537, Obras9191, Obras9385, Obras10342, Obras12982, Obras17500, Obras17566, Obras18660, Obras19202, Obras19481, Obras20420, Obras20432, Obras20659, Obras20998, Obras22358, Obras23412, Obras24115, Obras32341
Adrenomedullin	Vasodilation	Adrenomedullin	Obras13747, Obras14144, Obras18381, Obras19199
Savignygrin	Integrin α_IIb_β_3_	Kunitz-BPTI	Obras11352, Obras16076, Obras19477
Cystatin-2	Cathepsin B/C/H/L/S	Cystatin	Obras12401, Obras14164, Obras18092
Longistatin	Plasminogen	EF-hand	Obras12168, Obras12393
Serpin	Elastase/cathepsin G	Serpin	Obras4634, Obras5463, Obras5157, Obras5522, Obras5610, Obras5731, Obras5670, Obras5698, Obras6172, Obras6365
Carboxypeptidase inhibitor	Carboxypeptidase B	Inhibitor_I68	Obras19216

For the lipocalin family, a number of orthologs were found for proteins with known function, including leukotriene B_4_ (LTB_4_) scavengers (Mans and Ribeiro, 2008a). The LTB_4_ scavengers did not have the motifs conserved for complement C5 inhibition or thromboxane A_2_ scavenging and probably do not possess these functions (Nunn et al., 2005; Mans and Ribeiro, 2008a). Orthologs were also found for the biogenic amine scavengers and these orthologs possessed the biogenic amine-binding motif of the lower-binding site, while some also possessed the conserved amino acid residues involved in the upper-binding site of TSGP1 (Mans et al., 2008b). These orthologs therefore likely bind both histamine and serotonin. Orthologs for leukotriene C_4_ scavengers were also found (Mans and Ribeiro, 2008b).

In addition, an ortholog for the lipocalin (savicalin) was found. Savicalin was implicated in antimicrobial activity (Cheng et al., 2010). Other potential secretory antimicrobial orthologs present are defensins, microplusins, and trypsin inhibitor-like domains (Nakajima et al., 2001; Fogaça et al., 2004, 2006; Sasaki et al., 2008).

Four orthologs for adrenomedullin (vasodilation) were found in the transcriptome. Adrenomedullin was possibly acquired by soft ticks from the genus *Ornithodoros* by horizontal gene transfer from mammals and has thus far been found in *O. coriaceus*, *O. moubata*, *O. parkeri*, and *O. rostratus* (Iwanaga et al., 2014; Araujo et al., 2019). It would therefore seem as if the horizontal gene transfer occurred in the ancestral lineage to the *Pavlovskyella* and *Ornithodoros* (Figure 3). The presence of adrenomedullin in *O. brasiliensis* is therefore not surprising.

For members of the Kunitz-BPTI family, three orthologs of savignygrin were found. Single domain orthologs to the savignygrins were found that possessed the integrin RGD motif on the substrate binding presenting loop of the BPTI fold (Mans et al., 2002a). These inhibitors target the fibrinogen receptor integrin α_IIb_β_3_ and inhibit platelet aggregation. Orthologs have also been found in *A. monolakensis* suggesting that these inhibitors were evolved in the ancestral argasid lineage (Mans et al., 2008c). A double-domain Kunitz-BPTI protein (Obras12157) with the same integrin RGD recognition motif located in the second Kunitz domain was also found. Such double Kunitz domain proteins were also observed in the transcriptomes of *O. coriaceus* and *O. parkeri* (Francischetti et al., 2008a,b). To date, no experimental evidence exists that these target integrin α_IIb_β_3_, but it is likely that they target an integrin. The savignygrins are the Kunitz-BPTI proteins with the highest coverage for this family, as may be expected for an inhibitor that targets highly abundant platelet receptors in the host (Mans, 2019). Of interest is that no orthologs were found for the thrombin inhibitors like monobin, ornithodorin, or savignin (van de Locht et al., 1996; Nienaber et al., 1999; Mans et al., 2002b, 2008c), or the fXa inhibitors (Waxman et al., 1990; Gaspar et al., 1996; Joubert et al., 1998). This was also observed for Nearctic tick species, such as *O. coriaceus* and *O. parkeri* (Francischetti et al., 2008a,b). It was suggested that fXa inhibitors evolved exclusively within the genus *Ornithodoros* while thrombin inhibitors evolved in the ancestral argasid lineage (Mans et al., 2008c). Absence of these inhibitors in other Ornithodorinae lineages may indicate gene losses probably associated with host preferences. Possible orthologs to longistatin may be involved in blood coagulation modulation by activating plasminogen and in the immune system by targeting the receptor for advanced glycation end products (Anisuzzaman et al., 2011, 2014). Another ortholog associated with fibrinolysis is carboxypeptidase inhibitor that targets plasma carboxypeptidase B (thrombin-activatable fibrinolysis inhibitor) leading to fibrinolysis (Arolas et al., 2005). As such, a number of inhibitors that targets the blood coagulation cascade are present.

Orthologs of cystatin-2 from *O. moubata* were also detected. These cystatins inhibit cathepsin L and S allowing for modulation of the inflammatory responses in the host (Grunclová et al., 2006; Salát et al., 2010). Orthologs of serpins were also found that may inhibit elastase and cathepsin G acting as immunomodulators and platelet aggregation inhibitors (Prevot et al., 2006; Chmelar et al., 2011). It should be noted that the orthologs to which functions could be ascribed comprise ~19% of all the secretory proteins found in the transcriptome, suggesting that numerous undescribed functions still exist for this tick species.

### Potential Proteins Involved in Tick Toxicoses of *O. brasiliensis*

The toxicoses and bite of *O. brasiliensis* are accompanied by pruritis, local edema and erythema, pain, and blisters, while histopathology of the feeding site indicates extensive subcutaneous edema and hemorrhage (Reck et al., 2013a, 2014). Possibly, salivary proteins that may contribute to this clinical outcome would include the clotting and platelet aggregation inhibitors that would induce hemorrhage. On the other hand, the LTB_4_ and LTC_4_ scavengers may inhibit edema and erythema (Mans and Ribeiro, 2008b). Additional contributors to edema and hemorrhage may be the secretory proteolytic enzymes found in the transcriptome that includes a cathepsin, two serine proteases, and 38 metalloproteases (astacin, gluzincin, and reprolysin). These may also impact in wound-healing processes leading to prolonged recovery times.

### Evolutionary Perspectives on the Salivary Gland Transcriptome of *O. brasiliensis*

The salivary gland transcriptome of *O. brasiliensis* shows a number of evolutionary features previously found for argasids. This includes the presence of the major salivary gland secretory protein families conserved in all ticks (Mans et al., 2008a), as depicted in Figure 7. A number of orthologs for functions thus far conserved in argasids were also identified and include biogenic amine, leukotriene B_4_, and leukotriene C_4_ scavengers, as well as apyrase, savignygrin, and defensins (Mans and Ribeiro, 2008b). These functions are thus far conserved in all argasid species that feed on blood (Mans et al., 2016) and underscore their important role in blood-feeding. The only argasid salivary gland transcriptome sequenced to date that did not show these conserved features of protein families and conserved functions, were the salivary gland transcriptome for adult *A. delacruzi* (Ribeiro et al., 2012), which lacked all conserved features. This tick does not feed on a host in the adult phase and it is likely that this difference is due to differential expression in different life stages, and that these conserved features will be found in its blood-feeding stages. Since *Antricola* groups well within the Ornithodorinae, it may be expected that their lack of conserved protein families and functions in non-feeding phases is a derived trait. Conversely, the conserved protein families and functions as observed in *O. brasiliensis* and other argasids, indicate evolution of these traits in the last common ancestor to the Argasidae, or even the Ixodida (Mans et al., 2016).

## Conclusion

The current study reported 14,957 unique transcripts for the nymphal salivary gland transcriptome of *O. brasiliensis* considered to be of high quality. The transcriptome is enriched with secretory proteins with high abundance that belongs to well-characterized secretory protein families. Several orthologs could be identified of experimentally verified functions, while the data indicated that numerous functions remain to be discovered. Some of the secretory proteins identified in the transcriptome could also be linked to the clinical disease syndrome caused by *O. brasiliensis* and opens new avenues to identify the proteins involved in toxicoses.

## Data Availability Statement

The datasets presented in this study can be found in online repositories. The names of the repository/repositories and accession number(s) can be found at https://www.ncbi.nlm.nih.gov/genbank/, SRR14139641.

## Author Contributions

JR conceived the study, supplied the material, analyzed the data, and wrote the primary manuscript. AW supplied the material, analyzed the data, and critically reviewed the manuscript. BD supplied the material and critically reviewed the manuscript. RP, MC, and JF performed the experiments, analyzed the data, and critically reviewed the manuscript. BM conceived the study, performed the experiments, analyzed the data, and wrote the primary manuscript. All authors contributed to the article and approved the submitted version.

## Conflict of Interest

The authors declare that the research was conducted in the absence of any commercial or financial relationships that could be construed as a potential conflict of interest.

## Publisher’s Note

All claims expressed in this article are solely those of the authors and do not necessarily represent those of their affiliated organizations, or those of the publisher, the editors and the reviewers. Any product that may be evaluated in this article, or claim that may be made by its manufacturer, is not guaranteed or endorsed by the publisher.

## References

[ref1] Altschul S. F. Gish W. Miller W. Myers E. W. Lipman D. J. (1993). Basic local alignment search tool. J. Mol. Biol. 215, 403–410. 10.1006/jmbi.1990.9999, PMID: 2231712

[ref2] Anisuzzaman Islam M. K. Alim M. A. Miyoshi T. Hatta T. Yamaji K.. (2011). Longistatin, a plasminogen activator, is key to the availability of blood-meals for ixodid ticks. PLoS Pathog. 7:e1001312. 10.1371/journal.ppat.1001312, PMID: 21423674 PMC3053353

[ref3] Anisuzzaman H. T. Miyoshi T. Matsubayashi M. Islam M. K. Alim M. A. Anas M. A.. (2014). Longistatin in tick saliva blocks advanced glycation end-product receptor activation. J. Clin. Invest. 124, 4429–4444. 10.1172/jci74917, PMID: 25401185 PMC4191044

[ref301] Antunes S. Couto J. Ferrolho J. Sanches G. S. Merino Charrez J. O. De la Cruz Hernández N.. (2019). Transcriptome and proteome response of *Rhipicephalus annulatus* tick vector to *Babesia bigemina* infection. Front. Physiol. 10:318. 10.3389/fphys.2019.00318, PMID: 31001128 PMC6454348

[ref4] Araujo R. N. Silva N. C. S. Mendes-Sousa A. Paim R. Costa G. C. A. Dias L. R.. (2019). RNA-seq analysis of the salivary glands and midgut of the Argasid tick *Ornithodoros rostratus*. Sci. Rep. 9:6764. 10.1038/s41598-019-42899-z, PMID: 31043627 PMC6494864

[ref5] Arolas J. L. Lorenzo J. Rovira A. Castellà J. Aviles F. X. Sommerhoff C. P. (2005). A carboxypeptidase inhibitor from the tick *Rhipicephalus bursa*: isolation, cDNA cloning, recombinant expression, and characterization. J. Biol. Chem. 280, 3441–3448. 10.1074/jbc.M411086200, PMID: 15561703

[ref302] Bensaoud C. Nishiyama M. Y. Jr. Ben Hamda C. Lichtenstein F. Castro de Oliveira U. Faria F.. (2018). De novo assembly and annotation of *Hyalomma dromedarii* tick (Acari: Ixodidae) sialotranscriptome with regard to gender differences in gene expression. Parasit. Vectors 11:314. 10.1186/s13071-018-2874-9, PMID: 29793520 PMC5968504

[ref6] Bernt M. Donath A. Jühling F. Externbrink F. Florentz C. Fritzsch G.. (2013). MITOS: improved de novo metazoan mitochondrial genome annotation. Mol. Phylogenet. Evol. 69, 313–319. 10.1016/j.ympev.2012.08.023 22982435

[ref303] Bourret T. J. Boyle W. K. Zalud A. K. Valenzuela J. G. Oliveira F. Lopez J. E. (2019). The relapsing fever spirochete *Borrelia turicatae* persists in the highly oxidative environment of its soft-bodied tick vector. Cell. Microbiol. 21:e12987. 10.1111/cmi.12987, PMID: 30489694 PMC6454574

[ref7] Braz G. R. Coelho H. S. Masuda H. Oliveira P. L. (1999). A missing metabolic pathway in the cattle tick *Boophilus microplus*. Curr. Biol. 9, 703–706. 10.1016/s0960-9822(99)80312-1, PMID: 10395540

[ref8] Burger T. D. Shao R. Labruna M. B. Barker S. C. (2014). Molecular phylogeny of soft ticks (Ixodida: Argasidae) inferred from mitochondrial genome and nuclear rRNA sequences. Ticks Tick Borne Dis. 5, 195–207. 10.1016/j.ttbdis.2013.10.009, PMID: 24378079

[ref9] Cheng P. H. Mans B. J. Neitz A. W. Gaspar A. R. (2010). Savicalin, a lipocalin from hemocytes of the soft tick, *Ornithodoros savignyi*. Exp. Appl. Acarol. 52, 313–326. 10.1007/s10493-010-9368-6, PMID: 20512614

[ref10] Chmelar J. Oliveira C. J. Rezacova P. Francischetti I. M. Kovarova Z. Pejler G.. (2011). A tick salivary protein targets cathepsin G and chymase and inhibits host inflammation and platelet aggregation. Blood 117, 736–744. 10.1182/blood-2010-06-293241, PMID: 20940421 PMC3031492

[ref11] Dall’Agnol B. Schott D. Padilha T. Antunes P. Souza U. A. Webster A.. (2019). Clinical findings associated with *Ornithodoros brasiliensis* tick parasitism in travelers, southern Brazil. Wilderness Environ. Med. 30, 437–440. 10.1016/j.wem.2019.06.012, PMID: 31685323

[ref12] de Castro M. H. de Klerk D. Pienaar R. Latif A. A. Rees D. J. Mans B. J. (2016). De novo assembly and annotation of the salivary gland transcriptome of *Rhipicephalus appendiculatus* male and female ticks during blood feeding. Ticks Tick Borne Dis. 7, 536–548. 10.1016/j.ttbdis.2016.01.014, PMID: 26830274

[ref304] de Castro M. H. de Klerk D. Pienaar R. Rees D. J. G. Mans B. J. (2017). Sialotranscriptomics of *Rhipicephalus zambeziensis* reveals intricate expression profiles of secretory proteins and suggests tight temporal transcriptional regulation during blood-feeding. Parasit. Vectors 10:384. 10.1186/s13071-017-2312-4, PMID: 28797301 PMC5553602

[ref505] Esteves E. Maruyama S. R. Kawahara R. Fujita A. Martins L. A. Righi A. A.. (2017). Analysis of the salivary gland transcriptome of unfed and partially fed *Amblyomma sculptum* ticks and descriptive proteome of the saliva. Front. Cell. Infect. Microbiol. 7:476. 10.3389/fcimb.2017.00476, PMID: 29209593 PMC5702332

[ref13] Fogaça A. C. Almeida I. C. Eberlin M. N. Tanaka A. S. Bulet P. Daffre S. (2006). Ixodidin, a novel antimicrobial peptide from the hemocytes of the cattle tick *Boophilus microplus* with inhibitory activity against serine proteinases. Peptides 27, 667–674. 10.1016/j.peptides.2005.07.013, PMID: 16191451

[ref14] Fogaça A. C. Lorenzini D. M. Kaku L. M. Esteves E. Bulet P. Daffre S. (2004). Cysteine-rich antimicrobial peptides of the cattle tick *Boophilus microplus*: isolation, structural characterization and tissue expression profile. Dev. Comp. Immunol. 28, 191–200. 10.1016/j.dci.2003.08.001, PMID: 14642886

[ref15] Francischetti I. M. Mans B. J. Meng Z. Gudderra N. Veenstra T. D. Pham V. M.. (2008b). An insight into the sialome of the soft tick, *Ornithodorus parkeri*. Insect Biochem. Mol. Biol. 38, 1–21. 10.1016/j.ibmb.2007.09.009, PMID: 18070662 PMC2233652

[ref16] Francischetti I. M. Meng Z. Mans B. J. Gudderra N. Hall M. Veenstra T. D.. (2008a). An insight into the salivary transcriptome and proteome of the soft tick and vector of epizootic bovine abortion, *Ornithodoros coriaceus*. J. Proteome 71, 493–512. 10.1016/j.jprot.2008.07.006, PMID: 18725333 PMC2617759

[ref17] Francischetti I. M. Sa-Nunes A. Mans B. J. Santos I. M. Ribeiro J. M. (2009). The role of saliva in tick feeding. Front. Biosci. (Landmark Ed) 14, 2051–2088. 10.2741/3363 19273185 PMC2785505

[ref306] Garcia G. R. Gardinassi L. G. Ribeiro J. M. Anatriello E. Ferreira B. R. Moreira H. N.. (2014). The sialotranscriptome of *Amblyomma triste*, *Amblyomma parvum* and *Amblyomma cajennense* ticks, uncovered by 454-based RNA-seq. Parasit. Vectors 7:430. 10.1186/1756-3305-7-430, PMID: 25201527 PMC4261526

[ref18] Gaspar A. R. Joubert A. M. Crause J. C. Neitz A. W. (1996). Isolation and characterization of an anticoagulant from the salivary glands of the tick, *Ornithodoros savignyi* (Acari: Argasidae). Exp. Appl. Acarol. 20, 583–598. 10.1007/BF00052809, PMID: 8952072

[ref19] Grunclová L. Horn M. Vancová M. Sojka D. Franta Z. Mares M.. (2006). Two secreted cystatins of the soft tick *Ornithodoros moubata*: differential expression pattern and inhibitory specificity. Biol. Chem. 387, 1635–1644. 10.1515/BC.2006.204, PMID: 17132111

[ref20] Guglielmone A. A. Robbins R. G. Apanaskevich D. A. Petney T. N. Estrada-Peña A. Horak I. G.. (2010). The Argasidae, Ixodidae and Nuttalliellidae (Acari: Ixodida) of the world: a list of valid species names. Zootaxa 2528, 1–28. 10.11646/zootaxa.2528.1.1.1

[ref21] Gutierrez J. M. Feizi A. Li S. Kallehauge T. B. Hefzi H. Grav L. M.. (2020). Genome-scale reconstructions of the mammalian secretory pathway predict metabolic costs and limitations of protein secretion. Nat. Commun. 11:68. 10.1038/s41467-019-13867-y, PMID: 31896772 PMC6940358

[ref22] Hoogstraal H. (1985). Argasid and nuttalliellid ticks as parasites and vectors. Adv. Parasitol. 24, 135–238. 10.1016/s0065-308x(08)60563-1, PMID: 3904345

[ref23] Iwanaga S. Isawa H. Yuda M. (2014). Horizontal gene transfer of a vertebrate vasodilatory hormone into ticks. Nat. Commun. 5;3373. 10.1038/ncomms4373, PMID: 24556716

[ref24] Joubert A. M. Louw A. I. Joubert F. Neitz A. W. (1998). Cloning, nucleotide sequence and expression of the gene encoding factor Xa inhibitor from the salivary glands of the tick, *Ornithodoros savignyi*. Exp. Appl. Acarol. 22, 603–619. 10.1023/a:1006198713791, PMID: 9890144

[ref25] Kall L. Krogh A. Sonnhammer E. L. (2004). A combined transmembrane topology and signal peptide prediction method. J. Mol. Biol. 338, 1027–1036. 10.1016/j.jmb.2004.03.016, PMID: 15111065

[ref26] Kanehisa M. Sato Y. Kawashima M. Furumichi M. Tanabe M. (2016a). KEGG as a reference resource for gene and protein annotation. Nucleic Acids Res. 44, D457–D462. 10.1093/nar/gkv1070 26476454 PMC4702792

[ref27] Kanehisa M. Sato Y. Morishima K. (2016b). BlastKOALA and GhostKOALA: KEGG tools for functional characterization of genome and metagenome sequences. J. Mol. Biol. 428, 726–731. 10.1016/j.jmb.2015.11.006 26585406

[ref307] Karim S. Singh P. Ribeiro J. M. (2011). A deep insight into the sialotranscriptome of the gulf coast tick, *Amblyomma maculatum*. PLoS One 6:e28525. 10.1371/journal.pone.0028525, PMID: 22216098 PMC3244413

[ref308] Karim S. Ribeiro J. M. (2015). An insight into the sialome of the Lone Star tick, *Amblyomma americanum*, with a glimpse on its time dependent gene expression. PLoS One 10:e0131292. 10.1371/journal.pone.0131292, PMID: 26131772 PMC4489193

[ref28] Katoh K. Standley D. M. (2013). MAFFT multiple sequence alignment software version 7: improvements in performance and usability. Mol. Biol. Evol. 30, 772–780. 10.1093/molbev/mst010 23329690 PMC3603318

[ref29] Krogh A. Larsson B. von Heijne G. Sonnhammer E. L. (2001). Predicting transmembrane protein topology with a hidden Markov model: application to complete genomes. J. Mol. Biol. 305, 567–580. 10.1006/jmbi.2000.4315 11152613

[ref30] Li W. Godzik A. (2006). Cd-hit: a fast program for clustering and comparing large sets of protein or nucleotide sequences. Bioinformatics 22, 1658–1659. 10.1093/bioinformatics/btl158 16731699

[ref31] Mans B. J. (2019). Chemical equilibrium at the tick-host feeding interface: a critical examination of biological relevance in hematophagous behavior. Front. Physiol. 10:530. 10.3389/fphys.2019.00530 31118903 PMC6504839

[ref32] Mans B. J. (2020). Quantitative visions of reality at the tick-host interface: biochemistry, genomics, proteomics, and transcriptomics as measures of complete inventories of the tick sialoverse. Front. Cell. Infect. Microbiol. 10:574405. 10.3389/fcimb.2020.574405 33042874 PMC7517725

[ref33] Mans B. J. Andersen J. F. Francischetti I. M. Valenzuela J. G. Schwan T. G. Pham V. M.. (2008a). Comparative sialomics between hard and soft ticks: implications for the evolution of blood-feeding behavior. Insect Biochem. Mol. Biol. 38, 42–58. 10.1016/j.ibmb.2007.09.003 18070664 PMC2211429

[ref34] Mans B. J. Andersen J. F. Schwan T. G. Ribeiro J. M. (2008c). Characterization of anti-hemostatic factors in the argasid, *Argas monolakensis*: implications for the evolution of blood-feeding in the soft tick family. Insect Biochem. Mol. Biol. 38, 22–41. 10.1016/j.ibmb.2007.09.002 18070663 PMC4274796

[ref35] Mans B. J. de Castro M. H. Pienaar R. de Klerk D. Gaven P. Genu S.. (2016). Ancestral reconstruction of tick lineages. Ticks Tick Borne Dis. 7, 509–535. 10.1016/j.ttbdis.2016.02.002 26868413

[ref36] Mans B. J. de Klerk D. Pienaar R. de Castro M. H. Latif A. A. (2015). Next-generation sequencing as means to retrieve tick systematic markers, with the focus on *Nuttalliella namaqua* (Ixodoidea: Nuttalliellidae). Ticks Tick Borne Dis. 6, 450–462. 10.1016/j.ttbdis.2015.03.013 25936274

[ref37] Mans B. J. Featherston J. Kvas M. Pillay K. A. de Klerk D. G. Pienaar R.. (2019). Argasid and ixodid systematics: implications for soft tick evolution and systematics, with a new argasid species list. Ticks Tick Borne Dis. 10, 219–240. 10.1016/j.ttbdis.2018.09.010 30309738

[ref38] Mans B. J. Gasper A. R. Louw A. I. Neitz A. W. (1998). Purification and characterization of apyrase from the tick, *Ornithodoros savignyi*. Comp. Biochem. Physiol. B Biochem. Mol. Biol. 120, 617–624. 10.1016/s0305-0491(98)10061-5 14598857

[ref39] Mans B. J. Kelava S. Pienaar R. Featherston J. de Castro M. H. Quetglas J.. (2021). Nuclear (18S-28S rRNA) and mitochondrial genome markers of *Carios* (*Carios*) *vespertilionis* (Argasidae) support *Carios* Latreille, 1796 as a lineage embedded in the Ornithodorinae: re-classification of the *Carios* sensu Klompen and Oliver (1993) clade into its respective subgenera. Ticks Tick Borne Dis 12:101688. 10.1016/j.ttbdis.2021.101688 33652332

[ref40] Mans B. J. Louw A. I. Neitz A. W. (2002a). Savignygrin, a platelet aggregation inhibitor from the soft tick *Ornithodoros savignyi*, presents the RGD integrin recognition motif on the Kunitz-BPTI fold. J. Biol. Chem. 277, 21371–21378. 10.1074/jbc.M112060200 11932256

[ref41] Mans B. J. Louw A. I. Neitz A. W. (2002b). Amino acid sequence and structure modeling of savignin, a thrombin inhibitor from the tick, *Ornithodoros savignyi*. Insect Biochem. Mol. Biol. 32, 821–828. 10.1016/s0965-1748(01)00169-2 12044499

[ref42] Mans B. J. Neitz A. W. (2004). Molecular crowding as a mechanism for tick secretory granule biogenesis. Insect Biochem. Mol. Biol. 34, 1187–1193. 10.1016/j.ibmb.2004.07.007 15522614

[ref43] Mans B. J. Ribeiro J. M. (2008a). Function, mechanism and evolution of the moubatin-clade of soft tick lipocalins. Insect Biochem. Mol. Biol. 38, 841–852. 10.1016/j.ibmb.2008.06.007 18694828 PMC2613973

[ref44] Mans B. J. Ribeiro J. M. (2008b). A novel clade of cysteinyl leukotriene scavengers in soft ticks. Insect Biochem. Mol. Biol. 38, 862–870. 10.1016/j.ibmb.2008.06.002 18675910 PMC2583325

[ref45] Mans B. J. Ribeiro J. M. Andersen J. F. (2008b). Structure, function, and evolution of biogenic amine-binding proteins in soft ticks. J. Biol. Chem. 283, 18721–18733. 10.1074/jbc.M800188200 18445596 PMC2441560

[ref46] Mans B. J. Venter J. D. Coons L. B. Louw A. I. Neitz A. W. (2004). A reassessment of argasid tick salivary gland ultrastructure from an immuno-cytochemical perspective. Exp. Appl. Acarol. 33, 119–129. 10.1023/b:appa.0000030012.47964.b3 15285144

[ref47] Minh B. Q. Schmidt H. A. Chernomor O. Schrempf D. Woodhams M. D. von Haeseler A.. (2020). IQ-TREE 2: new models and efficient methods for phylogenetic inference in the genomic era. Mol. Biol. Evol. 37, 1530–1534. 10.1093/molbev/msaa015 32011700 PMC7182206

[ref309] Moreira H. N. S. Barcelos R. M. Vidigal P. M. P. Klein R. C. Montandon C. E. Maciel T. E. F.. (2017). A deep insight into the whole transcriptome of midguts, ovaries and salivary glands of the *Amblyomma sculptum* tick. Parasitol. Int. 66, 64–73. 10.1016/j.parint.2016.10.011, PMID: 27789388

[ref48] Nakajima Y. van der Goes van Naters-Yasui A. Taylor D. Yamakawa M. (2001). Two isoforms of a member of the arthropod defensin family from the soft tick, *Ornithodoros moubata* (Acari: Argasidae). Insect Biochem. Mol. Biol. 31, 747–751. 10.1016/s0965-1748(01)00066-2 11378409

[ref49] Nienaber J. Gaspar A. R. Neitz A. W. (1999). Savignin, a potent thrombin inhibitor isolated from the salivary glands of the tick *Ornithodoros savignyi* (Acari: Argasidae). Exp. Parasitol. 93, 82–91. 10.1006/expr.1999.4448 10502470

[ref50] Nunn M. A. Sharma A. Paesen G. C. Adamson S. Lissina O. Willis A. C.. (2005). Complement inhibitor of C5 activation from the soft tick *Ornithodoros moubata*. J. Immunol. 174, 2084–2091. 10.4049/jimmunol.174.4.2084 15699138

[ref51] Oleaga A. Soriano B. Llorens C. Pérez-Sánchez R. (2021). Sialotranscriptomics of the argasid tick *Ornithodoros moubata* along the trophogonic cycle. PLoS Negl. Trop. Dis. 15:e0009105. 10.1371/journal.pntd.0009105 33544727 PMC7891743

[ref52] Pérez-Sánchez R. Carnero-Morán Á. Soriano B. Llorens C. Oleaga A. (2021). RNA-seq analysis and gene expression dynamics in the salivary glands of the argasid tick *Ornithodoros erraticus* along the trophogonic cycle. Parasit. Vectors 14:170. 10.1186/s13071-021-04671-z 33743776 PMC7980729

[ref53] Perner J. Sobotka R. Sima R. Konvickova J. Sojka D. Oliveira P. L.. (2016). Acquisition of exogenous haem is essential for tick reproduction. eLife 5:e12318. 10.7554/eLife.12318 26949258 PMC4821805

[ref54] Petersen T. N. Brunak S. von Heijne G. Nielsen H. (2011). SignalP 4.0: discriminating signal peptides from transmembrane regions. Nat. Methods 8, 785–786. 10.1038/nmeth.1701 21959131

[ref55] Pienaar R. de Klerk D. G. de Castro M. H. Featherston J. Mans B. J. (2021). De novo assembled salivary gland transcriptome and expression pattern analyses for *Rhipicephalus evertsi evertsi* Neuman, 1897 male and female ticks. Sci. Rep. 11:1642. 10.1038/s41598-020-80454-3 33452281 PMC7810686

[ref56] Prevot P. P. Adam B. Boudjeltia K. Z. Brossard M. Lins L. Cauchie P.. (2006). Anti-hemostatic effects of a serpin from the saliva of the tick *Ixodes ricinus*. J. Biol. Chem. 281, 26361–26369. 10.1074/jbc.M604197200 16672226

[ref57] Ramirez D. G. Landulfo G. A. Onofrio V. C. Simons S. M. Reck J. Martins J. R.. (2016). Laboratory life cycle of *Ornithodoros brasiliensis* (Acari: Argasidae): An endemic tick from southern Brazil. Ticks Tick Borne Dis. 7, 730–733. 10.1016/j.ttbdis.2016.03.001 26972686

[ref58] Reck J. Bandarra P. Pavarini S. Termignoni C. Driemeier D. Martins J. R.. (2014). Experimentally induced tick toxicosis in rats bitten by *Ornithodoros brasiliensis* (Chelicerata: Argasidae): a clinico-pathological characterization. Toxicon 88, 99–106. 10.1016/j.toxicon.2014.06.017 24973739

[ref59] Reck J. Marks F. S. Guimarães J. A. Termignoni C. Martins J. R. (2013a). Epidemiology of *Ornithodoros brasiliensis* (mouro tick) in the southern Brazilian highlands and the description of human and animal retrospective cases of tick parasitism. Ticks Tick Borne Dis. 4, 101–109. 10.1016/j.ttbdis.2012.09.004 23238249

[ref60] Reck J. Marks F. S. Termignoni C. Guimarães J. A. Martins J. R. (2013b). *Ornithodoros brasiliensis* (mouro tick) salivary gland homogenates inhibit in vivo wound healing and in vitro endothelial cell proliferation. Parasitol. Res. 112, 1749–1753. 10.1007/s00436-013-3333-3 23397378

[ref61] Reck J. Soares J. F. Termignoni C. Labruna M. B. Martins J. R. (2011). Tick toxicosis in a dog bitten by *Ornithodoros brasiliensis*. Vet. Clin. Pathol. 40, 356–360. 10.1111/j.1939-165X.2011.00338.x 21827517

[ref62] Ribeiro J. M. Alarcon-Chaidez F. Francischetti I. M. Mans B. J. Mather T. N. Valenzuela J. G.. (2006). An annotated catalog of salivary gland transcripts from *Ixodes scapularis* ticks. Insect Biochem. Mol. Biol. 36, 111–129. 10.1016/j.ibmb.2005.11.005 16431279

[ref63] Ribeiro J. M. Endris T. M. Endris R. (1991). Saliva of the soft tick, *Ornithodoros moubata*, contains anti-platelet and apyrase activities. Comp. Biochem. Physiol. A Comp. Physiol. 100, 109–112. 10.1016/0300-9629(91)90190-n 1682082

[ref64] Ribeiro J. M. Labruna M. B. Mans B. J. Maruyama S. R. Francischetti I. M. Barizon G. C.. (2012). The sialotranscriptome of *Antricola delacruzi* female ticks is compatible with non-hematophagous behavior and an alternative source of food. Insect Biochem. Mol. Biol. 42, 332–342. 10.1016/j.ibmb.2012.01.003 22306723 PMC3351099

[ref310] Ribeiro J. M. Slovák M. Francischetti I. M. (2017). An insight into the sialome of *Hyalomma excavatum*. Ticks Tick Borne Dis. 8, 201–207. 10.1016/j.ttbdis.2016.08.011, PMID: 28049606 PMC5248969

[ref65] Ribeiro J. M. C. Mans B. J. (2020). TickSialoFam (TSFam): A database that helps to classify tick salivary proteins, a review on tick salivary protein function and evolution, with considerations on the tick sialome switching phenomenon. Front. Cell. Infect. Microbiol. 10:374. 10.3389/fcimb.2020.00374 32850476 PMC7396615

[ref311] Rodriguez-Valle M. Moolhuijzen P. Barrero R. A. Ong C. T. Busch G. Karbanowicz T.. (2018). Transcriptome and toxin family analysis of the paralysis tick, *Ixodes holocyclus*. Int. J. Parasitol. 48, 71–82. 10.1016/j.ijpara.2017.07.007, PMID: 28989068

[ref66] Salát J. Paesen G. C. Rezácová P. Kotsyfakis M. Kovárová Z. Sanda M.. (2010). Crystal structure and functional characterization of an immunomodulatory salivary cystatin from the soft tick *Ornithodoros moubata*. Biochem. J. 429, 103–112. 10.1042/BJ20100280 20545626 PMC3523712

[ref67] Sasaki S. D. de Lima C. A. Lovato D. V. Juliano M. A. Torquato R. J. Tanaka A. S. (2008). BmSI-7, a novel subtilisin inhibitor from *Boophilus microplus*, with activity toward Pr1 proteases from the fungus *Metarhizium anisopliae*. Exp. Parasitol. 118, 214–220. 10.1016/j.exppara.2007.08.003 17889850

[ref312] Schwarz A. Tenzer S. Hackenberg M. Erhart J. Gerhold-Ay A. Mazur J.. (2014). A systems level analysis reveals transcriptomic and proteomic complexity in *Ixodes ricinus* midgut and salivary glands during early attachment and feeding. Mol. Cell Proteom. 13, 2725–2735. 10.1074/mcp.M114.039289, PMID: 25048707 PMC4188998

[ref313] Schwarz A. von Reumont B. M. Erhart J. Chagas A. C. Ribeiro J. M. Kotsyfakis M. (2013). De novo *Ixodes ricinus* salivary gland transcriptome analysis using two next-generation sequencing methodologies. FASEB J. 27, 4745–4756. 10.1096/fj.13-232140, PMID: 23964076 PMC3834774

[ref68] Simao F. A. Waterhouse R. M. Ioannidis P. Kriventseva E. V. Zdobnov E. M. (2015). BUSCO: assessing genome assembly and annotation completeness with single-copy orthologs. Bioinformatics 31, 3210–3212. 10.1093/bioinformatics/btv351 26059717

[ref69] Šimo L. Sonenshine D. E. Park Y. Žitňan D. (2014). “Nervous and sensory systems: structure, function, genomics and proteomics,” in Biology of ticks, 2nd Edn. Vol. 1. eds. Sonenshine D. E. Roe R. M. (New York: Oxford University Press), 309–367.

[ref70] Stutzer C. Mans B. J. Gaspar A. R. Neitz A. W. Maritz-Olivier C. (2009). *Ornithodoros savignyi*: soft tick apyrase belongs to the 5'-nucleotidase family. Exp. Parasitol. 122, 318–327. 10.1016/j.exppara.2009.04.007, PMID: 19393241

[ref314] Tan A. W. Francischetti I. M. Slovak M. Kini R. M. Ribeiro J. M. (2015). Sexual differences in the sialomes of the zebra tick. Rhipicephalus pulchellus. J. Proteomics 117, 120–144. 10.1016/j.jprot.2014.12.014, PMID: 25576852 PMC4374903

[ref71] van de Locht A. Stubbs M. T. Bode W. Friedrich T. Bollschweiler C. Höffken W.. (1996). The ornithodorin-thrombin crystal structure, a key to the TAP enigma? EMBO J. 15, 6011–6017. 10.1002/j.1460-2075.1996.tb00989.x 8947023 PMC452422

[ref72] Waxman L. Smith D. E. Arcuri K. E. Vlasuk G. P. (1990). Tick anticoagulant peptide (TAP) is a novel inhibitor of blood coagulation factor Xa. Science 248, 593–596. 10.1126/science.2333510 2333510

